# P-330. BE-XPLORE: Barriers and Facilitators to Engaging Transgender and Gender-Diverse People in HIV and STI Research

**DOI:** 10.1093/ofid/ofaf695.549

**Published:** 2026-01-11

**Authors:** Rosalind Byrd, Abigail LeCates, Sarah Wishloff, Shericia Campbell, Athena Sherman, Cassie Grimsley Ackerley

**Affiliations:** Emory University, Atlanta, GA; Emory University School of Medicine, Atlanta, Georgia; Emory University, Atlanta, GA; Emory University, Atlanta, GA; Emory University, Atlanta, GA; Emory University, Atlanta, GA

## Abstract

**Background:**

Transgender and gender diverse (TGD) people often experience discrimination and stigma that increases their vulnerability to HIV and other STIs. Though prevalence is disproportionately high in these communities, some groups remain underrepresented in prevention research. We sought to identify barriers and facilitators to participation of TGD people in HIV/STI prevention research.

**Methods:**

Our mixed-methods approach, used online surveys and virtual semi-structured, in-depth interviews to identify prior experiences of and perceptions of research conducted within TGD community. Survey-takers were stratified into 4 groups: transmasculine (TM), transfeminine (TF), non-binary assigned female at birth (NB-AFAB), and non-binary assigned male at birth (NB-AMAB). 10 participants from each identity-group were interviewed. The interviews were transcribed, coded, and thematically analyzed.

**Results:**

141 surveys were completed -- 31 TF, 36 TM, 50 NB-AMAB, and 24 NB-AMAB. Ages ranged between 18 and 75 years old (median 28). There was diverse representation of racial/ethnic groups [White (79.4%), Black, African, or African American (19.9%), Hispanic/Latinx (8.5%), Asian (7.8%), and multiracial (14.2%)] and sexual orientations [queer (60%), bisexual (28%), and pansexual (26%), and straight (9%)]. Most described themselves as interested in HIV research (70%) or STI research (72%). Most common motivations were compensation or access to healthcare resources (medical care, medications, or testing). Concerns regarding invasive procedures, novel medications, and time strain were the most common barriers. Fair compensation and inclusive protocols were characteristics likely to draw participation.

**Conclusion:**

Identities and experiences within the TGD community are diverse. To reduce the impact of HIV and other STIs within this community, researchers must work to increase their representation in HIV/STI research by addressing individual and structural barriers, while prioritizing incentives that align with their needs and values.

**Disclosures:**

All Authors: No reported disclosures

